# Examination of Gender Stereotypes and Norms in Health-Related Content Posted to Snapchat Discover Channels: Qualitative Content Analysis

**DOI:** 10.2196/15330

**Published:** 2020-03-20

**Authors:** Kelsea LeBeau, Cary Carr, Mark Hart

**Affiliations:** 1 College of Public Health and Health Professions University of Florida Gainesville, FL United States

**Keywords:** social media, online social networking, health behavior, sexual health, social norms, gender, gender role, mobile applications

## Abstract

**Background:**

Snapchat has seen one of the most rapid, and unprecedented, growths in the history of social networking sites and social media with 3 billion Snapchats sent daily. In 2015, Snapchat introduced a new feature, Snapchat Discover, providing a unique way for publishers, such as magazines, to connect their content to Snapchat users.

**Objective:**

This study aimed to evaluate qualitatively the health-related content distributed among male-focused and female-focused Discover channels and to determine whether differences exist between the content posted to these channels.

**Methods:**

Magazine Discover channels with male and female target audiences were identified based on the magazine’s claimed audience and a search of Snapchat Discover’s magazine publishers, resulting in the selection of two male-focused and two female-focused channels. Stories were collected daily from each of the selected channels during a 4-week period. Using the constant comparative method, 406 Discover stories were collected and analyzed.

**Results:**

Differences in health content coverage existed between male- and female-focused channels. General health stories from male channels comprised 7.5% (10/134) of total stories compared with 22.8% (62/272) for female channels. Sexual health stories from male channels comprised 3.0% (4/134) of total stories compared with 18.8% (51/272) for female channels. Moreover, female-focused channels’ content was more comprehensive. Female audiences were portrayed as being health information seekers, concerned with sexual health and male satisfaction, primarily responsible for contraception and pregnancy prevention, and less informed about sex. Male audiences were portrayed as being less likely to seek health information, obsessed with and driven by sex, and less concerned with sexual health.

**Conclusions:**

Understanding the content shared to social media is important, especially when considering the implications content may have for behavior. In terms of content, these findings suggest Discover channels appear to promote gender stereotypes and norms for health and sexual health through the information posted.

## Introduction

### Snapchat Overview

Technology has become a major part of everyday life for both men and women, providing almost immediate and unlimited access to mass media, social networking sites (SNS), and social media platforms [1]. Although several popular SNS and social media platforms have been around for a while (eg, Facebook and Instagram), newer platforms have been gaining in popularity. One such platform is Snapchat (Snap, Inc), a social media app, which was founded in September 2011 and has become increasingly popular among smartphone users. Among those aged 18 to 34 years, Snapchat is now the third most popular app after Facebook and Instagram [2].

The growth of Snapchat has been one of the most rapid, and unprecedented, in the history of SNS and social media [3]. Snapchat had an estimated growth of approximately 90 to 100 million users from 2012 to 2015 [4,5]. As of 2018, there were roughly 3 billion Snapchats sent daily, 80 million daily active users in the United States, and 190 million daily active users worldwide [4]. Approximately 18% of US social media users use Snapchat, and the daily average time spent per user is 30 or more minutes [6]. Around 75% of Snapchat users are younger than 34 years old, and 90% of Snapchat users are between the ages of 13 and 24 years [6,7]. In addition, Snapchat has a disproportionate number of female users compared with male users, with roughly 70% of Snapchat users being female [6].

Snapchat is a mobile photo messaging and multimedia sharing app allowing users to send *snaps* consisting of photos or videos to people of their choosing. Ephemerality, the concept of something lasting temporarily, is a key feature differentiating Snapchat from other social media and makes the platform appealing to users [8]. Snapchat users can choose to send a snap with a specified time limit (between one and 10 seconds) or with no time limit (the snap does not disappear until you touch the screen again), offering users a more private and intimate form of communication compared with other social media [2].

### Snapchat Discover

In 2015, Snapchat introduced a new feature, Snapchat Discover, which is used by many Snapchatters [9]. In 2016, Snapchat reported more than 60 million people viewing its Discover platform each month [10]. The Snapchat Discover feature allows users to explore stories from various editorial teams on a daily basis. Essentially, Snapchat Discover is a network of media partners—ranging from National Geographic to Cosmopolitan—in which each partner has its own media channel where they can post articles, videos, and stories for their mobile audiences and subscribers [11]. Many channels are hosted by publishers that also have outside magazines, websites, or newspapers (eg, Cosmopolitan, National Geographic, BuzzFeed, and the Wall Street Journal).

Discover stories posted by publishers have similar transient features as personal snaps. Depending on the channel’s frequency of posting, stories can be available for as little as 24 hours to as long as a few days. Users are able to share stories with friends, but beyond that there is no way for a user to interact directly with a Discover story (ie, no commenting, liking, or direct saving).

### Gender Stereotypes, Gender Norms, and Social Media

Gender stereotypes and norms are common in our society [12,13] and are often endorsed across multiple media platforms, including SNS and social media [14-16]. These stereotypes and norms are usually constructed according to cultural values and practices, as well as cultural definitions of masculinity and femininity [12,14,17,18]. They tend to shape the perceptions of acceptable behaviors for men and women [18-21]. Masculinity is commonly associated with being strong, tough, aggressive, independent, and self-reliant. Masculine attributes include having and exercising power and control; the denial of weakness or vulnerability; participating in risky activities or behaviors; sexual infidelity or being less interested in committed, monogamous relationships; and avoiding open expression of emotions [12-14,17,19-21]. In contrast, femininity is commonly associated with being nurturing, affectionate, emotional, and sentimental; avoiding conflict; sexual fidelity; being more acquiescent, submissive, and dependent than males; and investing in physical appearance [12-15,20,21]. The stereotypes and norms endorsed by media and society also shape beliefs with regard to appropriate and expected health behaviors for males and females, including general health and sexual health [18,19,22-24]. In line with stereotypes for masculinity and femininity, being concerned with health, or seeking medical advice about general or sexual health, is seen as a feminine quality and something men do not need to engage in [12,17,25].

Pressure to conform to gender stereotypes and norms can have implications for health. In fact, certain behaviors considered normative for a particular gender have been shown to be associated with health consequences [12,18,26]. The impact gender stereotypes and norms have on health behaviors differ for men and women, with men often engaging in risky health behaviors and women often experiencing limitations in their ability to engage in behaviors that allow them to take control of their health [18]. Furthermore, the perpetuation of gender stereotypes and norms has been shown to influence sexual behaviors and beliefs and reinforce a sexual double standard [19,27].

In general, media sources are saturated with gendered messages that reinforce stereotypes and norms. Exposure to media and internet representations of gender have been shown to influence the endorsement of stereotypical expectations of men and women [16,27,28]. With the growing popularity and evolution of social media, however, it is unclear whether newer media platforms (eg, Snapchat) are reproducing gendered messages or are moving away from stereotypical representations of gender. As there is a limited amount of research in this area, it is important to explore whether gender stereotypes and norms are being reproduced on newer social media platforms.

### Review of Snapchat Research

Although its use has become increasingly prevalent [3], research has not kept pace with Snapchat’s rapid emergence. Research interests generally center on the use of Snapchat, often focusing on businesses and using Snapchat for brand building. Some research has begun to investigate the effects Snapchat has on interpersonal relationships [1,2]; the uses sought and gratifications obtained through Snapchat [5]; the influence Snapchat has on the social, emotional, and psychological experiences of its users [8]; the impact of social media use on psychosocial functioning during early adolescence [29]; the deterrent mechanisms associated with Snapchat use while driving [30]; the patterns of use on Snapchat [3]; and users’ perceptions of different aspects of Snapchat [31]. The focus of the majority of research studies is on the user’s interaction with Snapchat and not Snapchat’s interactions with its users.

### Objectives

A research area receiving little, if any, attention is an examination of newer Snapchat features that allow Snapchat to interact with its users, such as its Discover channels. Snapchat is able to disseminate a variety of information (such as news, current events, and health information) through these channels to an audience who often receives this type of information from SNS and social media platforms [32,33]. In a day and age in which health, sexual health, and gender norms are consistently being questioned and transformed, Snapchat has the opportunity to convey content in a new way that is both relevant and transformative to its audience.

To the researchers’ knowledge, no current research has examined the health content shared to Snapchat Discover channels. More specifically, no research has investigated the health content being shared to Discover channels as it relates to gender stereotypes and norms. Thus, an investigation of Discover channels is warranted to provide a better understanding of the content being posted with regard to health and gender. The purpose of this research study, then, was to evaluate qualitatively the general health and sexual health content posted to Discover channels. Further, this study intended to determine whether differences exist between the content posted to male-focused and female-focused channels. The study was guided by the following two research questions:

1. What health content, related to both general health and sexual health, is being shared to Snapchat Discover channels?
2. How does the content vary from male-focused channels to female-focused channels?

## Methods

### Selection of Discover Channels

Women’s and men’s magazines, in partnership with Snapchat, have created their own Discover channels and have become publishers on this platform. Magazines often have a stated gendered audience on their respective websites and cater their content to attract this audience [34]. Furthermore, magazines are often specifically labeled as either women’s magazines or men’s magazines, separating the type of magazine by gender [34,35]. It is reasonable, then, to assume that Discover channels affiliated with women’s and men’s magazines would have a similar target audience as the magazine’s specified audience outside of its Snapchat domain (posting content catered to either males or females). For this reason, magazine Snapchat publishers were chosen because of the ability to determine their gendered audience.

### Data Collection

Discover channels were selected from the Discover section on the Snapchat mobile app. To help sift through Discover channels on Snapchat and ensure a gendered audience could be identified, channels were selected according to a set of predetermined inclusion and exclusion criteria. Inclusion criteria were as follows: The channel was a designated publisher with its own publisher stories (content publishers created in partnership with Snapchat); the channel was affiliated with an existing magazine, and the affiliated magazine had information about the gender of their target audience; and an equal number of channels with a primary focus on a male audience and with a primary focus on a female audience had to be selected. Exclusion criteria were as follows: The channel could not be an influencer, and if information about the affiliated magazine’s audience was not available then the channel would not be used. The Snapchat Discover page on the mobile app was searched according to these criteria, and four publisher channels were selected: *GQ*, *Esquire*, *Cosmopolitan*, and *SELF*. These four channels were selected because they were the best available options meeting the inclusion and exclusion criteria at the time of data collection.

The target audiences for the four channels were determined according to their associated magazine website. *GQ* (formerly *Gentlemen’s Quarterly*) states it is the “premier men’s magazine” with the latest tips and advice for men [36]. *Esquire*’s website explains, “it’s a magazine for men…it is, and has been for nearly seventy years, a magazine about the interests, the curiosity, the passions, of men” [37]. *Cosmopolitan* is described as the “best-selling young women’s magazine in the U.S.” [38]. The *SELF* magazine website reads, “SELF Magazine: Women’s Workouts, Health Advice & Beauty Tips,” demonstrating a predominantly female audience.

Owing to the transient nature of Snapchat, data collection occurred daily. Each Discover channel updated their stories on a different schedule; therefore, data had to be collected and posts had to be saved every day. *Cosmopolitan* was the only channel that updated its stories every 24 hours; it was imperative data collection occurred the day it was posted, or the content would disappear without an alternative way to retrieve it. *GQ*, *Esquire*, and *SELF* all posted on different schedules: *GQ* posted stories two times a week (every Sunday and Wednesday), *Esquire* posted two times a week (every Tuesday and Friday), and *SELF* posted three times a week (every Wednesday, Saturday, and Monday).

It is important to note that saving content from Snapchat was challenging for this study. At the time of data collection, there was no direct way to download or save stories from the app, and stories were available only for a limited amount of time. Several months after the data collection for this study was completed, Snapchat released an update which made stories available for a longer period of time. This update made it possible for Discover stories to be revisited, creating more opportunities for data collection. The update has implications for future research on Discover channels because it would make the data collection process easier.

Data were collected from each of the four Snapchat channels during a specified 4-week period from January 21, 2018, to February 17, 2018. All titles and headlines for stories, with descriptions of what each story entailed, were documented. Screenshots of all stories specifically relevant to general health and sexual health were captured to assist with analyses. For this study, stories were considered related to general health if they mentioned physical, mental, spiritual, or emotional health and well-being. Stories were considered related to sexual health if they mentioned sexual activity, contraception or birth control, reproductive health or hygiene, sexual harassment, or sex-related items (ie, sex positions or sex toys). A total of 406 Discover stories (N=406) were collected and subsequently analyzed using the constant comparative method.

### Data Analysis

The constant comparative method was used for the qualitative analysis of the collected Snapchat data [39-42]. The constant comparative method is an iterative process of analyzing qualitative data, involving recoding and recategorizing data as they present themselves and creating new categories when codes do not fit into existing ones. It ensures the systematic comparison of all data with all other data in the data set [42] and is used to reduce data into manageable units and coded information [39-41]. An inductive approach to data analysis was used in which the coding of data was performed without using a pre-existing coding frame. This allowed for an organic exploration of themes as they emerged from the data. In this study, the codes, categories, and themes were directly drawn from the Snapchat data set; no preconceived codes or categories were used to inform the analysis process.

The constant comparative method has been increasingly utilized in SNS and social media research. Hart et al [43] used the method to categorize public health professionals’ tweets to evaluate how public health professionals are using Twitter as a platform to further the mission of public health. Fox and Moreland [44] used constant comparison to explore users’ negative emotional experiences within Facebook. In another study, Fox et al [45] examined the effects of Facebook on romantic relationships using the constant comparative method for analysis. In their study about social media use by physicians, Campbell et al [46] used the constant comparative method to understand the perceived risks and benefits of using social media as a health professional and the perceptions on social media use in health care. Finally, in their mixed method study, Bayer et al [8] used the constant comparative method to better understand college students’ social and emotional experiences on Snapchat.

The data analysis process began with an initial read through to gain an overall understanding of the data. Next, two researchers trained in qualitative research methods (KL and CC) independently open-coded all the Snapchat stories, sorting similar stories into categories. Open coding has been defined as “the process of breaking down, examining, comparing, conceptualizing, and categorizing data” [39-41]. Once initial codes were identified through open coding, comparative analyses were independently conducted within and across Snapchat stories using axial coding [41]. This allowed the researchers to refine the coding structure and group codes into higher level categories [41]. Then, the two researchers collectively identified major themes of the grouped data. Following the development of major themes, the researchers utilized selective coding to compare all stories with their major theme, using constant comparison to refine and sort stories to the most fitting theme. In addition, results were quantified by counting the number of units of data (stories) in each theme and subtheme, considering this information an objective measure to help summarize the results and guide interpretation of the findings.

All stories collected during the data collection period (N=406) were sorted into subthemes and themes through the constant comparative process; there were no stories considered *miscellaneous* or *other* that were not placed under one of the major themes. The researchers coded each story to only one theme or subtheme. Although a story may have related to more than one theme or subtheme, the theme or subtheme with which the story most related was the theme or subtheme that the story was coded under. If there was any disagreement between the two trained researchers, they discussed the topic of the story until an agreement was made. In addition, a third-party individual was available to help with disagreements.

## Results

### Discover Channels

The data collection process yielded 406 Snapchat Discover stories (N=406), all of which were included in the final analysis. Of the 406 stories, 61 (15.0%) came from *GQ*, 73 (18.0%) came from *Esquire*, 191 (47.0%) came from *Cosmopolitan*, and 81 (20.0%) came from *SELF*. *Cosmopolitan* stories made up almost half of the total stories collected. This is likely because it was the only channel that posted new content every day, whereas the other three channels posted stories two or three times per week.

*GQ* was one of the male-focused Discover channels used for comparison in this study. From the analysis, 16 subthemes were coded into five main themes: entertainment, health, fashion, dating and relationships, and grooming (see Multimedia Appendix 1). The theme with the most stories was fashion (27/61, 44%), with entertainment coming in second (13/61, 21%). This is not surprising given *GQ* magazine emphasizes fashion and style for men. The theme with the least number of stories was health (5/61, 8%). *GQ* was also the channel with the least number of stories overall compared with the other channels.

*Esquire* was the other male-focused Discover channel used for comparison in this study. There were 13 subthemes coded into five main themes: entertainment, health, sexual orientation, dating and relationships, and grooming (see Multimedia Appendix 1). The theme with the most stories was entertainment, comprising more than half (42/73, 58%) of all the stories posted by this publisher. The theme with the least number of stories was dating and relationships (2/73, 3%). The health theme was second to last, accounting for 8% (6/73) of stories.

*Cosmopolitan* was one of the female-focused Discover channels used for comparison in this study. *Cosmopolitan* had the most stories of all the channels analyzed, as well as the most themes and subthemes. There were 28 subthemes coded into seven main themes: entertainment, health, sex and relationships, appearances, politics, food and beverages, and home and home goods (see Multimedia Appendix 1). Themes with the most stories were entertainment (56/191, 29.3%), sex and relationships (52/191, 27.2%), and health (35/191, 18.3%). The themes with the least stories were home and home goods (9/191, 4.7%) and politics (5/191, 2.6%).

*SELF* was the second female-focused Discover channel used for comparison in this study. *SELF* had the second largest number of stories out of the four channels. As seen in Multimedia Appendix 1, *SELF* had 17 subthemes coded into six main themes: entertainment, health, fitness, sexual and reproductive health, dating and relationships, and appearance. Of the six major themes, the theme with the most stories was health (28/81, 35%), followed by fitness (15/81, 19%). This was the only channel that had a health theme with the most stories which is likely because *SELF* is billed as a health magazine. The theme with the least stories was dating and relationships (3/81, 4%).

### Health Content Comparison

The male-focused channels had few stories with general health content. When combined together, the male-focused channels had a total of 10 health stories, accounting for only 7.5% (10/134) of the total stories among the two channels (Table 1). The female-focused channels had a total of 62 stories with health content, accounting for 22.8% (62/272; approximately three times as many health stories as the male-focused channels) of the total stories among the two channels (Table 1).

**Table 1:** Number and percentages of general health stories from male- and female-focused Discover channels.

Discover channels		General health stories, n (%^a^)
**Male-focused channels (n=134)**		
	Combined	10 (7.5)
	*GQ^b^*	4 (3.0)
	*Esquire*	6 (4.5)
**Female-focused channels (n=272)**		
	Combined	62 (22.8)
	*Cosmopolitan*	19 (7.0)
	*SELF*	43 (15.8)

^a^Percentage of health stories from the combined total stories.

^b^GQ: Gentlemen Quarterly.

**Figure 1:**
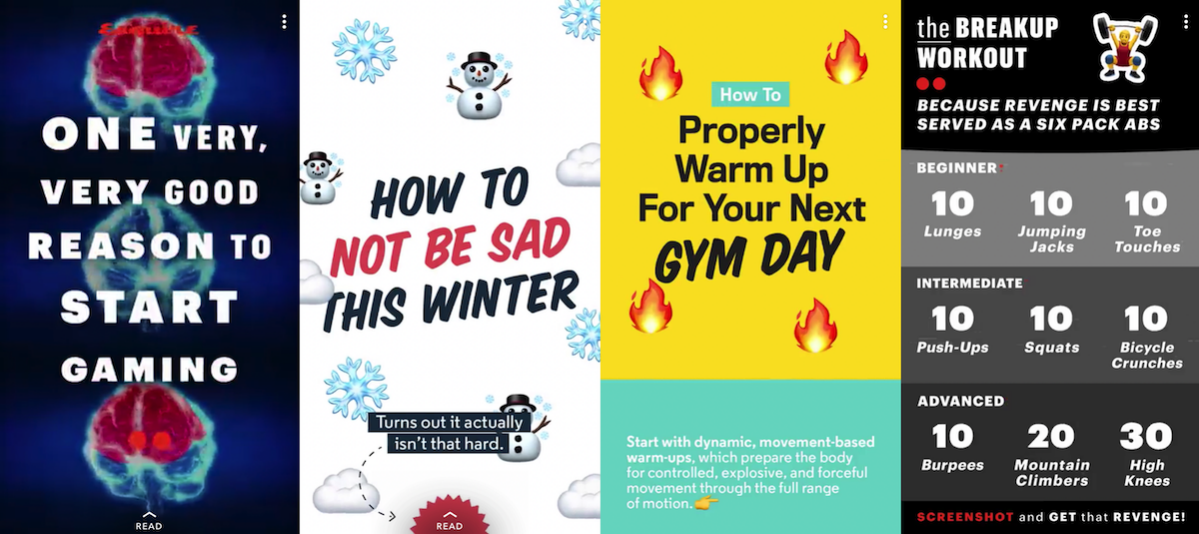
Examples of general health topics posted to male-focused Discover channels.

Differences in subthemes and general health content coverage existed between the male- and female-focused channels. The scope of general health content was greater for female-focused channels compared with male-focused channels, with female-focused channels covering a much wider range of topics (Table 2). Moreover, the health content addressed by male-focused channels covered most of their health topics only once. For example, the male-focused channels posted only one story about video games and health titled *One very, very good reason to start gaming*, which discussed the link found by researchers between video games and a reduced risk of Alzheimer disease (Figure 1). The male-focused channels also only posted one story about mental health titled *How to: Not be sad this winter*, which discussed the signs, symptoms, and treatment of seasonal affective disorder (Figure 1). The female-focused channels, however, often posted multiple separate stories about the same general health topic.

**Table 2:** Health content subthemes of male- and female-focused general health stories.

Gendered audience		Subthemes
**Male-focused channels**		
	*GQ^a^*	Exercise, mental health, and food
	*Esquire*	Exercise, video games and health, and health podcast recommendations
**Female-focused channels**		
	*Cosmopolitan*	Exercise, health information and advice, skin care, and emotional health
	*SELF*	Health information and advice, physical health, diet, exercise, exercise equipment, and recovery

^a^GQ: Gentlemen Quarterly.

**Figure 2:**
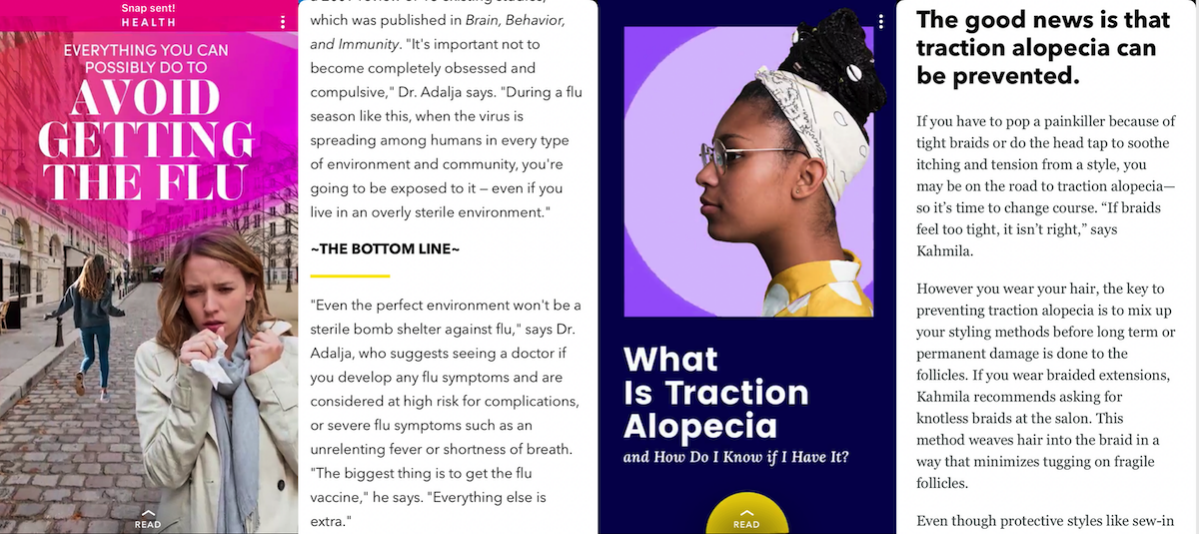
Examples of general health topics posted to female-focused Discover channels.

The male-focused channels were more general and less extensive in their approach to providing health content. A majority of the posted stories included only one or two brief paragraphs or a short list describing the health topic, including *How to Properly Warm Up for Your Next Gym Day* or *The Breakup Workout...Because Revenge is Best Served as a Six Pack of Abs* (Figure 1). In contrast, the general health content delivered by the female-focused channels posted more specific and descriptive health content. The channels’ stories offered tangible advice and in-depth explanations about the posted health topic, such as *Everything You Can Possibly Do To Avoid Getting the Flu*, *7 Weird Signs You’re Not Drinking Enough Water*, and *What Is Traction Alopecia and How Do I Know if I Have It?* (Figure 2).

### Sexual Health Content Comparison

The male-focused channels posted less stories covering sexual health content than the female-focused channels. Male-focused channels posted a total of four sexual health stories, comprising 3.0% (4/134) of the total stories among the two male-focused channels. The four sexual health stories only came from the *GQ*; *Esquire* did not post any stories with sexual health content (Table 3). The female-focused channels posted a total of 51 sexual health stories, comprising 18.8% (51/272) of the total stories among the two channels (Table 3). More than half of the sexual health posts came from *Cosmopolitan*. The female-focused channels also covered a larger variety of topics. The male-focused channels had two main subthemes addressing sexual health, whereas the female-focused channels had a number of subthemes addressing sexual health (Table 4).

**Table 3:** Number and percentages of sexual health stories from male- and female-focused Discover channels.

Discover channels			Sexual health stories, n (%^a^)
**Male-focused channels (n=134)**			
	Combined	4 (3.0)	
	*GQ^b^*	4 (3.0)	
	*Esquire*	0 (0.0)	
**Female-focused channels (n=272)**			
	Combined	51 (18.8)	
	*Cosmopolitan*	35 (12.9)	
	*SELF*	16 (5.9)	

^a^Percentage of health stories from the combined total stories.

^b^GQ: Gentlemen Quarterly.

**Table 4:** Sexual health content subthemes of male- and female-focused sexual health stories.

Gendered audience		Subthemes
**Male-focused channels**		
	*GQ^a^*	Dating and sex
	*Esquire*	None
**Female-focused channels**		
	*Cosmopolitan*	Pregnancy, sexual and reproductive health, sexual assault and harassment, dating, sex toys, and sex secrets
	*SELF*	Birth control, feminine hygiene, reproductive health, women’s rights, and dating

^a^GQ: Gentlemen Quarterly.

**Figure 3:**
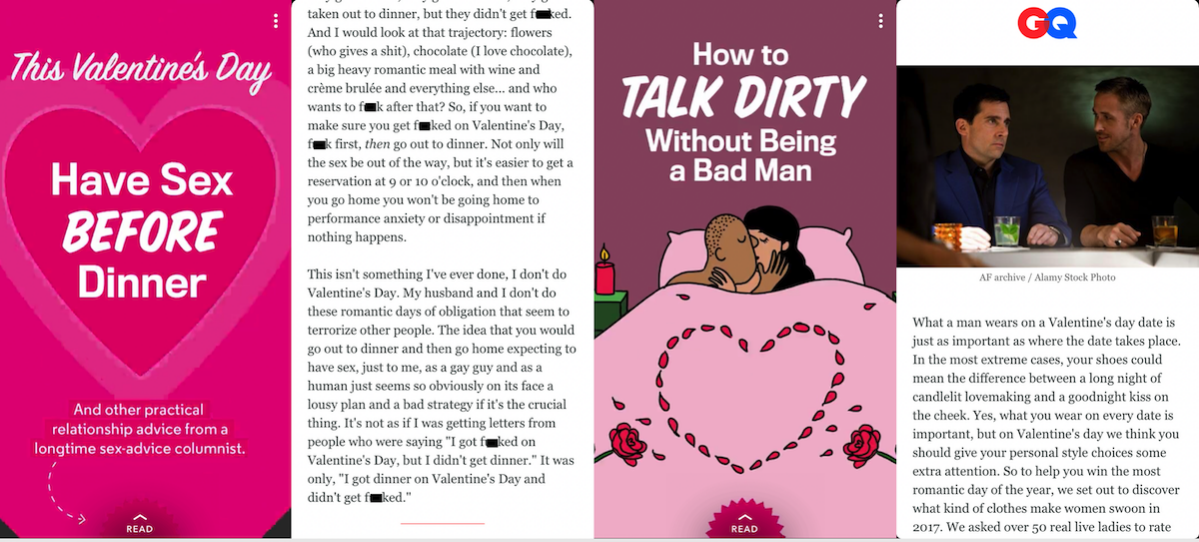
Examples of sexual health topics posted to male-focused Discover channels.

Although there were only four stories for the male-focused channels, apparent differences in content emerged. The male-focused channels did not post any sex-related stories that specifically included sexual health information; rather, they posted about sexual activity and sexual success. For example, one of the stories titled *This Valentine’s Day Have Sex Before Dinner* discussed engaging in sexual activity before going out to dinner on Valentine’s Day to guarantee you successfully engage in sexual activity. The man giving the advice wrote, “if you want to make sure you get f****ed on Valentine’s day, f*** first, *then* go out to dinner” (Figure 3). Another story titled *Never Play Acoustic Guitar for a Woman* told men to “never play guitar for someone you’re trying to sleep with” because it is not seductive and might be a turn off. The third story titled *What Should You Wear on Valentine’s Day?* gave advice about what a man should wear to avoid a “goodnight kiss on the cheek” and successfully ensure a “long night of candlelit lovemaking” (Figure 3).

**Figure 4:**
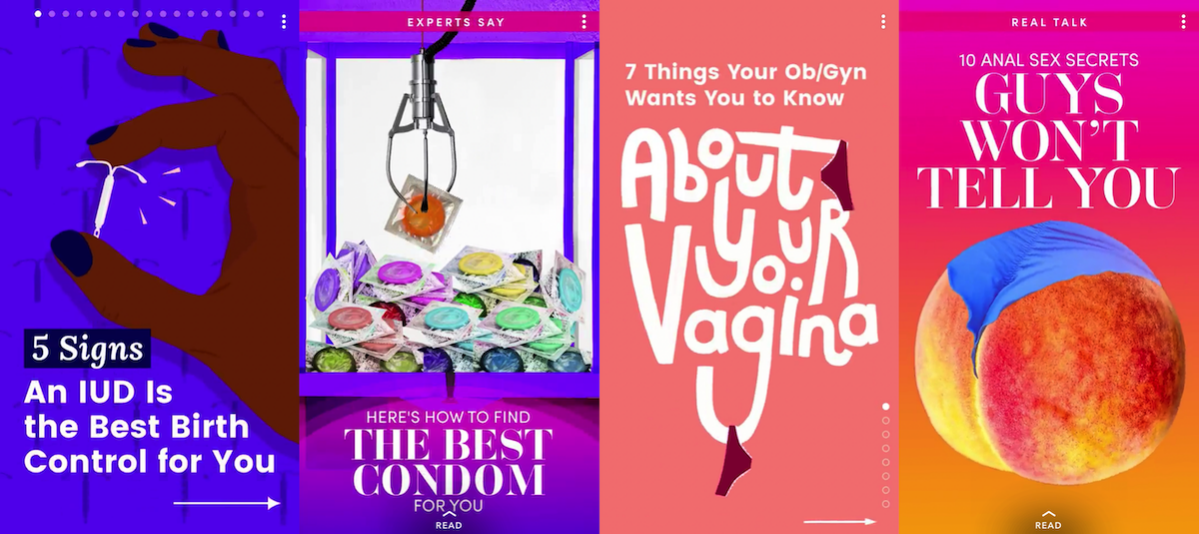
Examples of sexual health topics posted to female-focused Discover channels.

The female-focused channels posted stories with a variety of sex-related topics, many of which included sexual health-related information, and were the only channels to post stories about contraception. Stories about contraception included *7 Best Places to Get Free Condoms*, *How to Find the Best Condom for You*, *5 Signs an IUD is the Best Birth Control for You*, and *Here are the Signs an IUD Isn’t Right for You* (Figure 4). Interestingly, the female-focused channels did mention males providing condoms or preparing for sexual intercourse by bringing condoms in a couple of their stories. The male-focused channels made no mention of contraceptive use in any of their stories about sex.

The female-focused channels also posted sexual health information about reproductive health (such as female internal and external genitalia) and feminine hygiene. This information was often presented in the form of advice or recommendations from reputable sources (eg, doctors, specialists, and experts). Examples include stories such as *7 Things Your OB/GYN Wants You to Know About Your Vagina*, *11 Things You Need to Know About Your Vulva*, *Is It Normal to Have a Really Long Menstrual Cycle?*, and *Keeping Your Vagina Clean* (Figure 4). The male-focused channels did not offer similar information about male reproductive health or hygiene in their posted stories.

Another difference in the sexual health content across female- and male-focused channels was the way in which they were framed. Stories posted to female-focused channels often made it seem as if men possess secrets about sexual intercourse and sexual activities that they do not want women to know. Examples of these stories include *9 Sixty-Nine Secrets Guys Won’t Tell You*, *10 Anal Sex Secrets Guys Won’t Tell You*, and *10 Sex Toy Secrets Guys Won’t Tell You* (Figure 4). The focus of these stories was often about what men think or how men feel without mentioning the same for women. In contrast, as stated previously, the main focus of the stories from the male-focused channels was on setting up men for sexual success and sexual pleasure.

## Discussion

### Principal Findings

This study sought to examine the general health and sexual health content being posted to Snapchat Discover channels, as well as to determine whether differences exist between the content posted based on the gender-specific audiences. In examining the content of Discover channels, this preliminary study moves beyond the traditional focus on the effect of users’ interactions with main Snapchat features to explore a different feature that offers a variety of health information to users through some of its publisher channels. Compared with the male-focused channels, female-focused channels had more stories offering general health–related and sexual health–related content. The scope of their general health and sexual health content was greater than that of the male-focused channels, and the number of stories addressing each general health and sexual health topic was greater for female-focused than male-focused channels. The findings from this study demonstrate that the analyzed Snapchat Discover channels reflect and reiterate stereotypical conceptions of gender through the content posted. If users are adopting behaviors learned from content posted to these channels, then it is possible that users not only are being exposed to this content but also might be engaging in behaviors that reinforce gender stereotypes and norms for general health and sexual health.

### General Health Content and Promotion of Gender Stereotypes

A focus of this study was to investigate the general health content being shared to Discover channels and the differences in content posted to male- and female-focused channels. It is evident from the analysis that target audiences are perceived differently by their respective publishers with regard to their overall health behaviors and health information–seeking behaviors. Stories posted to the male-focused channels were more superficial in their general health content. These channels mostly posted health information with the intent of providing ways to make you appear more attractive, not necessarily with the intent of providing helpful information to enhance or maintain your overall health. This reflects the gender stereotype that participating in health-promoting behaviors is perceived as less masculine [12,17,18]. In contrast, the female-focused channels established a sense of agency and control for their audience over their health and well-being. These channels addressed more aspects of overall health with an emphasis on prevention, self-care, healthy habits, and taking ownership of your health.

These findings are in line with research that has documented significant gender differences in health behaviors [12,17,18]. In fact, gender has been found to be one of the most important factors that influences health-related behavior, with women generally adopting healthier behavior practices than men [12]. Traditional masculine ideals endorse the gender stereotype that men should be strong, independent, and self-reliant, and therefore do not need to practice health-seeking behaviors [12,17,18]. Whether intentionally or not, by reiterating this gender stereotype through content posted (or, in some cases, not posted), it is possible that these male-focused channels are influencing social norms among their target audience. The content shared to these channels is promoting the idea that health behaviors should be more important to one gender than the other, and that health-related behaviors are defined by one’s gender. There is a need to undo the stereotypes and norms that make health behaviors typical of a single gender, and instead inclusive of all genders. Social media could take steps toward this by representing health behaviors in new ways and posting about health behaviors to both male- and female-focused Snapchat channels.

### Sexual Health Content and Promotion of Gender Stereotypes

The findings suggest that Discover channels also promote sexual health gender stereotypes and norms. There was a difference in the number of posts with sexual health–related content, with female-focused channels posting more content. This difference suggests that sexual relationships and sexual health are primarily topics for women to concern themselves with and topics men do not have to worry about. Thus, women are seen as wanting and needing sexual health information, whereas similar information is seen as unimportant or unnecessary for men, or knowledge men already possess.

In line with findings from previous research [19,35], when sex-related topics were posted to the male-focused channels, the primary focus was on men’s sexual freedom and sexual success in heterosexual relationships. All of the stories with sexual content focused on how to successfully ensure you have sex with a woman. Men were told how to dress attractively to have sex with their date, how to plan their evening around having sex, how to appropriately *talk dirty*, and what not to do to have sex with a woman. These stories endorse the stereotypes that link being obsessed with and driven by sex to masculinity [12,22,35], undermining the idea that men should be interested and involved in their sexual health. They also promote the stereotype that women serve to provide sex to men on men’s terms [14,35].

Furthermore, the lack of sexual health–related information among male-focused channels suggests that casual sexual intercourse and activities are normal for men without any sexual health concerns or responsibilities attached to them. The male-focused channels posted four stories specifically about having sex without any mention of contraception or sexual health. This contributes to the perception of sex as a carefree activity for men and reinforces the stereotype that safe sex practices (in heterosexual relationships) are primarily the responsibility of women [20,23,35]. Female-focused channels posted some content about condoms and birth control. Condoms were described to females as cost-effective forms of birth control that can protect against sexually transmitted infections (STIs) when used correctly. All condoms discussed were male condoms, with no mention of female condoms, which is interesting considering these channels have mainly female audiences. The presence of sexual health content on female-focused channels, and not on male-focused channels, reinforces the stereotype that women bear the responsibility for contraception and pregnancy prevention [22,35]. It is important to note that the responsibility for safe sex in heterosexual relationships often hinges on the woman’s ability to negotiate safe sex practices. Negotiations such as these require some degree of assertiveness, an attribute that is societally viewed as contradictory to femininity. This conflict between assertiveness and passivity complicates conversations about sexual safety for women [35]. Research has found that power imbalances in relationships make it difficult to plan to have safe sex, such as taking precautions to protect against STIs and pregnancy [24]. Thus, the findings also demonstrate that the stigma attached to women carrying condoms, in combination with the expectations for women to be responsible for pregnancy prevention, might put women in a difficult situation that could lead to negative consequences, such as STIs or unplanned pregnancies [19,24].

Moreover, the stories posted to female-focused channels often instructed women about how to understand men’s sexual interests and behaviors by disclosing the *secrets* men are keeping from them. Men are portrayed as possessing secrets about their own sexual behavior women want to know. Essentially, Snapchat stories are acting as portals that unlock access to secrets, so women can learn how men think and feel about sexual intercourse and activities, with the goal of enabling women to serve men’s desires more successfully. The same types of secrets are not shared through stories on the male-focused channels. This difference in content suggests an interesting power differential, aligning with similar findings from a study about sexual double standards [19,20]. Being portrayed as possessing all of the knowledge they need about sex puts men in a stereotypical masculine position of power and control [12,19,35]. It is possible that the female-focused channels are attempting to address this power differential by revealing the secrets men are keeping; however, the way the content is framed promotes the idea that men do, in fact, have more knowledge about sex than women, and women should be most concerned with providing male pleasure.

The amount of information about reproductive health and hygiene posted to the Discover channels also reinforced gender stereotypes and norms regarding sexual health. The female-focused channels posted several stories about female internal and external genitalia and appropriate feminine hygiene, whereas the male-focused channels posted no sexual health content about reproductive health and hygiene. This agrees with research that have found norms surrounding femininity encourage women to understand and examine their own bodies and recognize subtle signs of change [25]. Norms surrounding masculinity discourage men from understanding or examining their bodies [25]. Men tend to avoid discussing sexual health issues, which could be why male-focused channels also avoid posting stories containing this type of content. These channels may be providing a disservice to their male audiences by failing to post meaningful discussions that consider the role of masculinity in relation to sexual health.

### Limitations and Recommendations

This study is not without limitations. First, the data are subjective based on the researchers’ interpretations, as is the nature of qualitative research. The information gained from a qualitative process, however, provides a nuanced understanding into the content posted by Snapchat publishers for their gender-specific audiences. Another limitation of this study is that the data collection period was only 4 weeks long. It is possible that different themes would have emerged had the data collection period been longer. The limited number of stories and short data collection period reduce the explanatory power and generalizability of the findings. Future research examining Snapchat Discover stories should collect data for a longer period of time to enhance explanatory power and generalizability. In addition, there are limitations to the selected channels themselves. As the channels selected for this study had to be linked to magazines (based on the inclusion and exclusion criteria), the choices of channels were limited. The nature of the channels selected might not be the most representative or comparable with respect to audience and content. For example, *GQ* is often described as a style magazine and is likely to have a different male audience than other magazines. The same could be said for the audiences of *SELF* and *Cosmopolitan*. The content posted to these channels is likely to align with the purpose and target audience of the magazine. Thus, we cannot generalize these findings to all Discover channels on Snapchat. Future research could benefit by identifying Discover channels that have more comparable target audiences and focus mostly on health and sexual health content. It might also be important to consider the number of subscribers a channel has. This study did not select channels based on the number of subscribers; however, it is possible that the popularity of a channel might impact the content being posted and how much influence a channel could have on its users.

As social media continues to evolve, opportunities are presented for these platforms to conform with, challenge, or defy societal expectations and normative ideas [14]. Social media apps have the ability to break away from gender stereotypes and norms by changing the type of content they deliver, and the way relevant topics are discussed. Snapchat Discover channels, in particular, are capable of doing this by posting general health and sexual health content in a way that does not conform to gender stereotypes and norms. In doing so, these platforms can transform alongside their audiences who are slowly changing and building new meanings of health, gender, sexuality, and identity [34].

The authors have a few practical recommendations to aid these processes. First, social media publishers should acknowledge the pervasiveness of gender stereotypes and accept that these stereotypes might be biasing the content being posted. This could help to identify and correct such biases [28]. Second, social media publishers could work to educate themselves and their content curators about gender stereotypes [28]. This includes learning about the implications that reinforcing and endorsing stereotypes might have for consumers of the content. It is important for social media publishers to be more aware of the potential implications gendered messages can have for users, especially with regard to health and health behaviors. It is also important for social media platforms to bear responsibility for their content decisions. Third, social media publishers could seek feedback from members of a different gender before posting potential content. This could be in the form of encouraging equal representation of genders on content creation teams or hiring a gender expert to consult on content creation. Finally, social media publishers should work to create new understandings of how they view their gendered audiences. Publishers could then use their new understandings to generate content that incorporates more effective and inclusive gender portrayals.

### Conclusions

This study has provided a first look at the general health and sexual health content posted to Snapchat Discover channels. The findings suggest that publishers posting content to the male-focused channels view their audiences as less likely to seek health information than their female counterparts, as obsessed with and driven by sex, and as less concerned with sexual health. Publishers creating content for the female-focused channels view their audiences as health information seekers, as concerned with sexual health, as responsible for contraception and pregnancy prevention, and as less informed about sex than their male counterparts. The content posted to the male- and female-focused channels seems to be promoting gender stereotypes and responsibilities for health information seeking and for sexual health and sexual relationships. By posting relevant content in new ways that neither represents traditional femininity nor hegemonic masculinity, social media can help to slowly transform restrictive gender norms and socially constructed stereotypes.

## Appendix

Multimedia Appendix 1 Themes and underlying subthemes of Snapchat Discover channel stories.

## Abbreviations

SNS: social networking sites
STI: sexually transmitted infection
