# Supplementary material for: Examination of Gender Stereotypes and Norms in Health-Related Content Posted to Snapchat Discover Channels: Qualitative Content Analysis
Source: J Med Internet Res. 2020 Mar 20;22(3):e15330. doi: 10.2196/15330 (PMC7125437; doi:10.2196/15330)
Supplement: Multimedia Appendix 1 [file jmir_v22i3e15330_app1.docx]

## Appendix

| Discover Channel themes and underlying subthemes. | |
| --- | --- |
| **Themes** (count) | **Subthemes** (count) |
| ***GQ*** (5) | ***GQ*** (16) |
| Entertainment | Television, celebrities, music, movies |
| Health | Exercise, mental health, food |
| Fashion | Clothing, trends, shoes |
| Dating & Relationships | Dating, break-up, sex |
| Grooming | Skin care, tips, hair |
| ***Esquire*** (5) | ***Esquire*** (13) |
| Entertainment | Television, music, sports, video games, podcast recommendations |
| Health | Exercise, video games and health, health podcast recommendations |
| Sexual orientation | Olympics, coming out |
| Dating and Relationships | Break-up, dating |
| Grooming | Hair |
| ***Cosmopolitan*** (7) | ***Cosmopolitan*** (28) |
| Entertainment | Television, celebrities, movies, Olympics |
| Health | Exercise, health information and advice, skin care, pregnancy, Sexual and reproductive health, emotional health, sexual assault and harassment |
| Sex and Relationships | Sex toys, sex secrets, dating, cheating, marriage, friendship |
| Appearances | Hair, makeup, fashion |
| Politics | DACA, State of the Union, president |
| Food and Beverages | Alcohol, snacks, dessert |
| Home and Home goods | Home, décor |
| ***SELF*** (6) | ***SELF*** (17) |
| Entertainment | Celebrities, Olympics |
| Health | Physical health, health information and advice, diet, bathroom |
| Fitness | Exercise, recovery, equipment |
| Sexual and Reproductive Health | Birth control, feminine hygiene, reproductive health, women’s rights |
| Dating and Relationships | Dating |
| Appearance | Clothing, body image, beauty |
